# Malate as a key carbon source of leaf dark-respired CO_2_ across different environmental conditions in potato plants

**DOI:** 10.1093/jxb/erv279

**Published:** 2015-07-02

**Authors:** Marco M. Lehmann, Katja T. Rinne, Carola Blessing, Rolf T. W. Siegwolf, Nina Buchmann, Roland A. Werner

**Affiliations:** ^1^Laboratory of Atmospheric Chemistry, Paul Scherrer Institute (PSI), CH-5232 Villigen, Switzerland; ^2^Institute of Agricultural Sciences, ETH Zurich, Universitaetsstr. 2, CH-8092 Zurich, Switzerland

**Keywords:** Compound-specific isotope analysis (CSIA), drought, organic acids, plant respiration, stable carbon isotopes, sugars, temperature, tricarboxylic acid (TCA) cycle.

## Abstract

Dissimilation of carbon sources during plant respiration in support of metabolic processes results in the continuous release of CO_2_. The carbon isotopic composition of leaf dark-respired CO_2_ (i.e. *δ* ^*13*^ *C*_*R*_) shows daily enrichments up to 14.8‰ under different environmental conditions. However, the reasons for this ^13^C enrichment in leaf dark-respired CO_2_ are not fully understood, since daily changes in δ^13^C of putative leaf respiratory carbon sources (*δ* ^*13*^ *C*_*RS*_) are not yet clear. Thus, we exposed potato plants (*Solanum tuberosum*) to different temperature and soil moisture treatments. We determined *δ* ^*13*^ *C*_*R*_ with an in-tube incubation technique and *δ* ^*13*^ *C*_*RS*_ with compound-specific isotope analysis during a daily cycle. The highest *δ* ^*13*^ *C*_*RS*_ values were found in the organic acid malate under different environmental conditions, showing less negative values compared to *δ* ^*13*^ *C*_*R*_ (up to 5.2‰) and compared to *δ* ^*13*^ *C*_*RS*_ of soluble carbohydrates, citrate and starch (up to 8.8‰). Moreover, linear relationships between *δ* ^*13*^ *C*_*R*_ and *δ* ^*13*^ *C*_*RS*_ among different putative carbon sources were strongest for malate during daytime (r^2^=0.69, *P*≤0.001) and nighttime (r^2^=0.36, *P*≤0.001) under all environmental conditions. A multiple linear regression analysis revealed *δ* ^*13*^ *C*_*RS*_ of malate as the most important carbon source influencing *δ* ^*13*^ *C*_*R*_. Thus, our results strongly indicate malate as a key carbon source of ^13^C enriched dark-respired CO_2_ in potato plants, probably driven by an anapleurotic flux replenishing intermediates of the Krebs cycle.

## Highlight

Carbon isotope analyses revealed malate as a key carbon source of leaf dark-respired CO_2_ in potato plants under different temperature and soil moisture conditions during a daily cycle.

## Introduction

The investigation of plant respiration as a major process in plant biochemistry has expanded our understanding of carbon cycling in autotrophic organisms. Plants dissimilate carbon sources for the production of intermediates and reducing equivalents in support of metabolic processes, thereby continuously releasing CO_2_ via plant respiration (Hopkins, 2006). Leaf-respired CO_2_ is mainly derived from oxidative decarboxylation reactions catalysed by enzymes from the Krebs cycle (KC) and from interacting anabolic and catabolic reactions (Voet and Voet, 2011).

Using stable isotopes, the pathway of carbon can be traced from photosynthetic carbon fixation to respiratory carbon loss. On the one hand, C_3_ plants discriminate heavily against ^13^C due to photosynthetic isotope fractionation, leading to general ^13^C depletion in plant biomass of about 20‰ in comparison to atmospheric CO_2_ (Farquhar *et al.*, 1989). The exact magnitude of photosynthetic carbon isotope discrimination depends on the intercellular CO_2_ concentration (*C*_*i*_) in the substomatal cavity, which is regulated by other physiological parameters such as net assimilation rate (*A*_*n*_) and stomatal conductance (*g*_*s*_). Environmental conditions such as light, temperature, soil moisture, and air humidity will influence these parameters and with them the photosynthetic carbon isotope discrimination. On the other hand, the carbon isotopic composition of leaf dark-respired CO_2_ (i.e. *δ* ^*13*^ *C*_*R*_) has clearly been shown to be less negative than leaf metabolites in several plant species (Ghashghaie *et al.*, 2003; Bowling *et al.*, 2008; Werner and Gessler, 2011; Ghashghaie and Badeck, 2014). In a daily cycle, leaf dark-respired CO_2_ follows a progressive ^13^C enrichment during the day and a gradual ^13^C depletion during the course of the night (Hymus *et al.*, 2005; Prater *et al.*, 2006), resulting in a strong temporal variability of up to 14.8‰ (Barbour *et al.*, 2007; Werner *et al.*, 2009; Wegener *et al.*, 2010), which differs among functional groups (Priault *et al.*, 2009; Werner *et al.*, 2009).

*δ* ^*13*^ *C*_*R*_ is thereby linked to the carbon isotopic composition of putative leaf respiratory carbon sources (i.e. *δ* ^*13*^ *C*_*RS*_) such as carbohydrates (soluble mono- and di-saccharides, and starch) and organic acids. Previous studies showed that environmental drivers such as temperature and soil moisture influence *δ* ^*13*^ *C*_*R*_ and *δ* ^*13*^ *C*_*RS*_. More negative *δ* ^*13*^ *C*_*R*_ values with increasing temperature have been observed with short-term changes in leaf temperature during darkness in *Phaseolus vulgaris* (Tcherkez *et al.*, 2003), while long-term effects of higher temperatures on *δ* ^*13*^ *C*_*R*_ and *δ* ^*13*^ *C*_*RS*_ have not yet been investigated under controlled conditions. Other studies have demonstrated less negative *δ* ^*13*^ *C*_*R*_ and *δ* ^*13*^ *C*_*RS*_ values under dry conditions compared to those under wet conditions (Duranceau *et al.*, 1999; Ghashghaie *et al.*, 2001). Similar observations were made in field experiments (Sun *et al.*, 2009; Dubbert *et al.*, 2012). Conversely, more negative *δ* ^*13*^ *C*_*R*_ values have been found under dry conditions for Mediterranean trees and herbs such as *Quercus ilex* and *Tuberaria guttata* compared to those under wet conditions (Unger *et al.*, 2010), which have been explained with accompanied increases in temperatures and vapour pressure deficit. Nevertheless, the combined effects of temperature and soil moisture on *δ* ^*13*^ *C*_*R*_ and *δ* ^*13*^ *C*_*RS*_ under controlled conditions have yet to be tested.

Moreover, *δ* ^*13*^ *C*_*R*_ is determined by various post-photosynthetic carbon isotope fractionation processes at pivotal branching points in respiratory pathways, carbon isotope effects on enzymatic reactions, and changes in respiratory substrates (for a detailed review see Werner and Gessler, 2011). The ^13^C enrichment in leaf dark-respired CO_2_ itself is thought to be a result of fragmentation fractionation processes based on heterogeneous intramolecular carbon isotope distribution in respiratory carbon sources (Tcherkez *et al.*, 2004). For instance, C-3 and C-4 positions of glucose are known to be enriched in ^13^C compared to the other molecule positions due to an isotope effect of the aldolase reaction (Rossmann *et al.*, 1991; Gleixner and Schmidt, 1997). Breakdown of glucose during glycolysis produces pyruvate with a ^13^C enriched C-1 position (former C-3 and C-4 positions of glucose). Thereafter, the pyruvate dehydrogenase reaction (PDH) releases the C-1 position as ^13^C enriched CO_2_, whereas the more ^13^C depleted acetyl-CoA residue is used in the KC (Priault *et al.*, 2009; Werner and Gessler, 2011). Thus, a PDH dominated respiratory pathway may lead to ^13^C enrichment in leaf dark-respired CO_2_.

However, the knowledge about *δ* ^*13*^ *C*_*R*_ is often based on light-acclimated leaves, which have been transferred into darkness to allow respiratory measurements. This approach holds an unpreventable bias known as ‘light-enhanced dark respiration’ (LEDR), which needs to be taken into account when interpreting daytime *δ* ^*13*^ *C*_*R*_ values. LEDR is a short-term light-dark transition period, describing an increase in the amount of leaf dark-respired CO_2_ shortly upon darkening for about 20min, which depends on light intensity (Atkin *et al.*, 1998). On the one hand, LEDR may be influenced by reassembly of the KC, which is thought to be only partially active under light conditions (Tcherkez *et al.*, 2005; Sweetlove *et al.*, 2010; Werner and Gessler, 2011; Werner *et al.*, 2011). On the other hand, LEDR may be driven by a breakdown of a light-accumulated malate pool, causing ^13^C-enriched leaf dark-respired CO_2_ (Barbour *et al.*, 2007; Gessler *et al.*, 2009; Werner *et al.*, 2009; Barbour *et al.*, 2011; Werner and Gessler, 2011). Malate itself is also known to be ^13^C enriched compared to other carbon sources (Gleixner *et al.*, 1998; Ghashghaie *et al.*, 2001). The ^13^C enrichment in malate was attributed to an anapleurotic flux via the phosphoenolpyruvate carboxylase reaction (PEPC), which fixes ^13^C-enriched hydrogen carbonate and replenishes KC intermediates (Melzer and O’Leary, 1987; Savidge and Blair, 2004). Thus, a possible breakdown of malate by the mitochondrial malic enzyme reaction, or within the KC, may influence *δ* ^*13*^ *C*_*R*_ (Barbour *et al.*, 2007; Werner *et al.*, 2011). In addition, plants may also use to a certain extent more complex carbon sources such as lipids and proteins under severe environmental conditions or under prolonged darkness (Tcherkez *et al.*, 2003; Usadel *et al.*, 2008). However, the driving processes, the respiratory carbon sources, and the mechanisms causing changes in *δ* ^*13*^ *C*_*R*_ during day and night are not fully resolved thus far.

Hence, with this study we intend to assess two major research questions. What causes the high daily variations in *δ* ^*13*^ *C*_*R*_? How are *δ* ^*13*^ *C*_*R*_ and *δ* ^*13*^ *C*_*RS*_ influenced by temperature and soil moisture conditions? Our main objectives were (i) to analyse the relationship between *δ* ^*13*^ *C*_*R*_ and *δ* ^*13*^ *C*_*RS*_ values and (ii) to determine changes in *δ* ^*13*^ *C*_*R*_ and *δ* ^*13*^ *C*_*RS*_ values, as well as in concentrations of the putative carbon sources under different environmental conditions. Therefore, we exposed potato plants to different controlled temperature and soil moisture conditions and measured *δ* ^*13*^ *C*_*R*_ with an in-tube incubation technique, as well as *δ* ^*13*^ *C*_*RS*_ and concentrations of soluble carbohydrates, organic acids and starch from leaves with compound specific isotope analysis (CSIA) on a daily basis.

## Materials and methods

### Plant material

Potato plants (*Solanum tuberosum* L. cv. Annabell) were grown from tubers of the same size in 5 l pots filled with bark humus soil (Ökohum, Herrenhof, Switzerland) in a greenhouse, with average temperatures of 20/16°C and vapour pressure deficits (*VPD*) of about 0.9/0.4 kPa (day/night). The plants were exposed to a 16h daylight period supplemented by 400W sodium-lamps (Powertone Son-T Plus, Philips, Amsterdam, Netherlands). Forty days after planting, plants were transferred into walk-in climate chambers for acclimatization for 2 weeks. The 16h daylight in the climate chambers had an averaged photosynthetic photon flux density of ~400 µmol m^-2^ s^-1^ at leaf level, thus plants were not fully light-saturated. Before the treatment period, soil water status was optimal for at least 3 d after watering, while an individual plant consumed about 300ml water per day. 50ml of a 0.4% fertilizer solution (v/v, Gesal, Zürich, Switzerland) was applied twice to all plants during the whole experiment of 70 d.

Treatments were applied during the last 15 d of the experiment. Plants were exposed to high temperature (T_high_) of 28/23°C (day/night) and low temperature conditions (T_low_) of 22/17°C, at a *VPD* of about 0.9/0.35 kPa for both temperature treatments. Three climate chambers were used for replication of each temperature treatment. Within each climate chamber there were two soil-moisture treatments with nine plants each. Dry soil moisture conditions were kept constantly at 50–60% of the daily water consumption of each individual plant, determined by weighing the entire pots. Plants under wet conditions were kept at 100%.

The final sampling period lasted 32h during the last 2 d of the experiment, when dry soil conditions were established for both temperature treatments. Sampling was done on a daily basis every 2h (nighttime) or 4h (daytime). During sampling, individual plants had 3–6 ranks, with about four fully developed leaves per rank. Always the third-last fully developed leaf per rank was sampled at all points in time, but within 24h only one sample was taken from each individual plant to avoid any stress response induced by sampling. Sampled leaf material was immediately frozen in liquid nitrogen and stored at −80°C. Subsequently, the leaf material was freeze-dried and milled to powder by a steel ball mill (MM200, Retsch, Haan, Germany) for all further isotopic and biochemical analyses. In addition to leaf sampling, air CO_2_ samples from all six climate chambers were collected at the same points in time during the sampling period, showing a mean δ^13^C value of −12.2‰ and typical daily variations of SD ≤1.4‰; no differences between temperature treatments (*P*≥0.05) and points in time (*P*≥0.05; linear mixed effects model) were observed during the daily cycle.

### Physiological measurements and biomass determination

Several leaf physiological parameters were determined with an infrared gas analyser (LI-6400, LI-COR, Lincoln, Nebraska, USA), including net assimilation rate (*A*_*n*_), intercellular CO_2_ concentration (*C*_*i*_), and stomatal conductance (*g*_*s*_). All measurements were taken in the last 4h of the daylight phase. To monitor volumetric soil water content (*SWC*), up to three soil moisture sensors (EC-5 and logger Em5b, Decagon Devices, Pullman, USA) were installed for each treatment. Shortly after the sampling period, total plant biomass was harvested, oven-dried (at 60°C), and weighed. The fresh tuber weight and tuber count (number of potatoes) were determined.

### Carbon isotope and concentration analyses

δ^13^C values are expressed as described by Craig (1957) and modified by Coplen (2011):

$$\delta^{\text{13}}\text{C }\left(‰\right)={\text{R}}_{\text{sample}}/{\text{R}}_{\text{standard}}-\text{1}$$

where R_sample_ is the ^13^C/^12^C ratio of the sample material and R_standard_ is that of the international standard VPDB (Vienna Pee Dee Belemnite).

### Determination of δ^13^C_R_

The in-tube incubation technique was used for the collection of leaf dark-respired CO_2_ during daytime and nighttime (Werner *et al.*, 2007). A leaf was placed in a 12ml gas-tight exetainer (Labco, Lampeter, UK), which was immediately darkened with a lightproof casing to trigger leaf dark respiration. The tube was then flushed for 1min with synthetic air until a CO_2_-free atmosphere was established, which was monitored with an infrared gas analyser (LI-6262, LI-COR, Lincoln, Nebraska, USA). After an incubation time of 3min in darkness, an aliquot of dark-respired CO_2_ was transferred with a gas-tight syringe into a new exetainer filled with dry N_2_. *δ* ^*13*^ *C*_*R*_ values were determined with an IRMS, using a modified Gasbench II (Thermo Fisher, Bremen, Germany) connected to a Delta^plus^XP-IRMS, similar to Zeeman *et al.* (2008). The transfer of the CO_2_ sample into a new exetainer, as well as the IRMS measuring procedure, were both tested with air of known δ^13^C of CO_2_ to ensure no isotope fractionation had occurred. Measurement precision of a quality control standard (three standards per 24 samples) was SD≤0.1‰.

### Determination of δ^13^C in bulk leaves and leaf starch

Extraction of leaf starch was performed as described in previous studies (Wanek *et al.*, 2001; Goettlicher *et al.*, 2006; Richter *et al.*, 2009). Leaf starch was isolated from 50mg leaf material with methanol/chloroform/water (MCW, 12:5:3, v/v/v) at 70°C for 30min. Samples were centrifuged (10 000 ×*g*, 2min) and supernatants removed, while the leaf-starch-containing pellets were washed with MCW and deionized water and dried at room temperature (RT). Pellets were then re-suspended in water and boiled at 99°C for 15min to facilitate starch gelatinization. Subsequently, leaf starch was enzymatically digested with α-amylase (EC 3.2.1.1, Sigma-Aldrich, Buchs, Switzerland) at 85°C for 2h, and cleaned with centrifugation filters to remove enzymes (Vivaspin, Sartorius, Göttingen, Germany). To determine δ^13^C of bulk leaves (*δ* ^*13*^ *C*_*leaf*_) and starch, an elemental analyser (Flash EA 1112 Series) coupled to a Delta^plus^XP-IRMS was used (both Thermo Fisher, Bremen, Germany; Werner *et al.*, 1999). Measurements of samples, blanks, and reference material followed the identical treatment principle described by Werner and Brand (2001). The long-term precision of a quality control standard for all sequences was SD≤0.12‰.

### Isotopic and concentration analysis of soluble carbohydrates and organic acids

Water-soluble compounds were extracted from 100mg leaf material with water at 85°C for 30min, similar to Streit *et al.* (2013). Subsequently, soluble carbohydrates and organic acids were separated by ion-exchange chromatography (Wanek *et al.*, 2001; Goettlicher *et al.*, 2006; Richter *et al.*, 2009), using Dowex 50WX8 in H^+^-form and Dowex 1X8 in NaCOO^-^-form (both 100–200 mesh, Sigma-Aldrich, Buchs, Switzerland). To avoid clogging of the HPLC column by polyphenols, all samples designated for carbohydrate analyses were filtered with 100mg Sep-Pak C18 Vac RC Cartridges (Waters AG, Milford, Massachusetts, USA). Finally, all carbohydrate and organic acid samples were cleaned with 0.45 µm PTFE syringe filter (Infochroma AG, Zug, Switzerland) prior to HPLC measurements.

To determine *δ* ^*13*^ *C*_*RS*_ values and the concentrations of soluble carbohydrates and organic acids, a HPLC-IRMS system consisting of a high performance liquid chromatograph coupled to a Delta V Advantage IRMS by a LC IsoLink (all Thermo Fisher, Bremen, Germany) was used according to Krummen *et al.* (2004). Carbohydrates were separated on a 3×150mm anion-exchange column CarboPac PA20 (Dionex, Olten, Switzerland) using 2mM NaOH as the mobile phase and a flow speed of 250 µl min^-1^ (Boschker *et al.*, 2008; Rinne *et al.*, 2012). Low column temperature of 20°C was used to prevent isomerization of hexoses (Rinne *et al.*, 2012). This enabled chromatographic separation for sucrose and glucose, but fructose *δ* ^*13*^ *C*_*RS*_ and concentration measurements were affected by partial co-elution of fructose with other compounds. To correct *δ* ^*13*^ *C*_*RS*_ values and to calculate concentrations from the peak areas, interspersed standard solutions in a concentration range of 20–180ng C µl^-1^ were measured within each sequence. The measurement precision of *δ* ^*13*^ *C*_*RS*_ values in all carbohydrate standards was SD<0.5‰. Below a concentration of 60ng C µl^-1^, the precision of fructose standards was lower for certain batches, and therefore these results were excluded.

Organic acids were separated on a 4.6×300mm Allure Organic Acids column (Restek, Bellefonte, USA) at 5–10°C. The mobile phase was a 100mM monopotassium phosphate buffer (pH 3) with a flow speed of 500 μl min^-1^ (Hettmann *et al.*, 2005). The measurement precision of δ^13^C in organic acid standards was SD<0.4‰. Low citrate concentrations from T_low_ samples (<45ng C µl^-1^) impeded the analytical accuracy of the *δ* ^*13*^ *C*_*RS*_ values, therefore these samples were not taken into account.

All purification steps were verified for each batch of 24 samples using 2.5mg standard solutions of known δ^13^C (by EA-IRMS) for all carbohydrates and organic acids measured in this study. Differences between δ^13^C values before and after purification were generally ≤0.2‰, indicating no significant isotope fractionation for any standard. Mean recovery was 101±6% for fructose, 96±6% for glucose, 89±3% for sucrose, 91±3% for malate, and 86±3% for citrate.

### Determination of starch concentration

For the extraction of leaf starch for concentration analyses we used a modified method of Critchley *et al.* (2001). Leaf starch was isolated with 1.12M perchloric acid from 50mg leaf material at RT for 15min and centrifuged (10min, 3000 ×*g*, 4°C). The supernatant was removed and the leaf-starch-containing pellet was washed free from pigments with deionized water and ethanol. Pellets were then dried at RT, resuspended in water, and gelatinized. Subsequently, starch samples were enzymatically hydrolysed to glucose for 2h at 37°C with a solution mix of α-amylase (EC 3.2.1.1, Sigma-Aldrich, Buchs, Switzerland) and α-amyloglucosidase (EC 3.2.1.3, Roche, Rotkreuz, Switzerland) in 220mM sodium acetate buffer (pH 4.8). The glucose concentration was determined at 340nm with a 96-well microplate reader (EL×800, BioTek, Luzern, Switzerland) using a coupled enzymatic reaction (Hoch *et al.*, 2002). Potato starch was used as a standard. Glucose concentrations are expressed in molarity of starch monomers.

### Data analysis

R version 3.0.2 (R Core Team, 2013) was used for (multiple) linear regression analyses and linear mixed effects models (R package nlme). Models included fixed effects (temperature, soil moisture, sampling time) and random effects (climate chambers, individual plants). If applicable, δ^13^C values and concentrations were logarithmically transformed to ensure normal distribution. For the best-fit combination of the multiple linear regression analysis, variables were excluded if *P*≥0.05.

## Results

### Physiological parameters and biomass

**Fig. 1.**
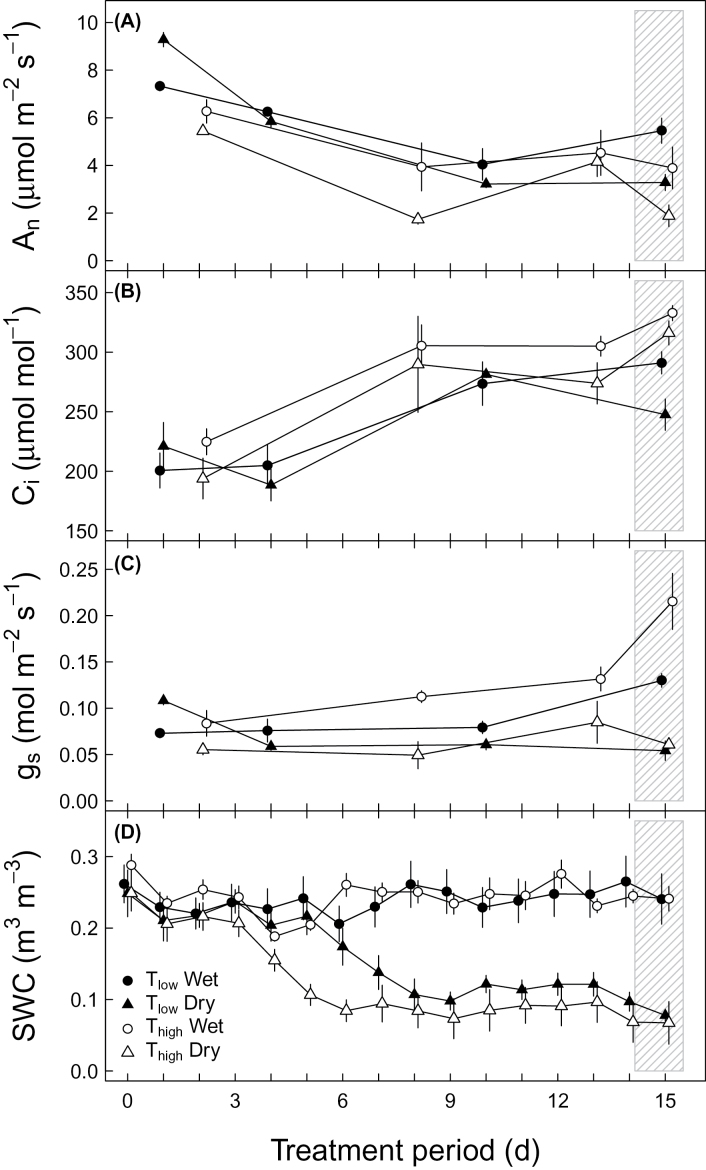
Physiological parameters under different environmental conditions during the treatment period: (A) net assimilation rate (*A*_*n*_, µmol m^-2^ s^-1^), (B) intercellular CO_2_ concentration (*C*_*i*_, µmol mol^-1^), (C) stomatal conductance (*g*_*s*_, mol m^-2^ s^-1^), (D) volumetric soil water content (*SWC*, m^3^/m^-3^). Potato plants were treated with a combination of T_low_ (low temperature; closed symbols), T_high_ (high temperature; open symbols), and wet (circles) or dry (triangles) conditions. Boxed areas indicate the sampling period. Means ±SE are given (*n*=3).

Physiological parameters (*A*_*n*_, *C*_*i*_, *g*_*s*_, and *SWC*) of potato plants exposed to four different treatments were monitored during the treatment period of 15 d (Fig. 1). The net assimilation rate declined during the treatment period under all four treatments (Fig. 1A). During the sampling period (Fig. 1A, day 15), *A*_*n*_ was significantly influenced by soil moisture (*P*=0.02, Table 1), with lowest values (1.9 µmol m^-2^ s^-1^) under *T*_*high*_ and dry conditions, and highest values (5.4 µmol m^-2^ s^-1^) under *T*_*low*_ and wet conditions, whereas the temperature influence on *A*_*n*_ was not significant (*P*=0.07, Table 1) but tended to cause lower *A*_*n*_ values under T_high_ than under T_low_ under both soil moisture conditions. The intercellular CO_2_ concentration increased during the treatment period for all four treatments (Fig. 1B). During the sampling period (Fig. 1B, day 15), *C*_*i*_ was independently influenced by temperature (*P*=0.012, Table 1) and soil moisture (*P*=0.01, Table 1), with lowest *C*_*i*_ (247.5 µmol mol^-1^) under *T*_*low*_ and dry conditions and highest *C*_*i*_ (332.8 µmol mol^-1^) under *T*_*high*_ and wet conditions. Stomatal conductance during the treatment period was lower under dry treatments compared to those under wet treatments (Fig. 1C). During the sampling period (Fig. 1C, day 15), *g*_*s*_ was significantly influenced by soil moisture (*P*≤0.001, Table 1), with lowest *g*_*s*_ (about 0.06mol m^-2^ s^-1^) in plants of both dry treatments and highest *g*_*s*_ (0.22mol m^-2^ s^-1^) in plants under T_high_ and wet conditions, whereas the temperature influence under wet conditions tended to cause higher *g*_*s*_ values under T_high_ than under T_low_. The volumetric soil water content was lower under dry conditions (~7–14%) compared to wet conditions (23–27.5%) for the last 9 d of the treatment period (Fig. 1D), including the sampling period (Fig. 1D, day 15), where *SWC* was significantly affected only by soil moisture treatments (*P*=0.002, Table 1). Generally, no significant interactions between temperature and soil moisture were observed for any parameter (Table 1). In addition, only soil moisture treatments affected plant biomass (*P*=0.008, Table 1) and tuber weight (*P*=0.023, Table 1) taken shortly after the sampling period, independent of temperature treatments. Highest values tended to be under *T*_*low*_ and wet conditions and lowest values under *T*_*high*_ and dry conditions (Tables 1, 2), indicating different stress levels created by the four treatments.

**Table 1.** Environmental influences on physiological parameters Results of linear mixed effects models testing the effects of temperature (low, high) and soil moisture (wet, dry) on physiological parameters (*A_n_*, net assimilation rate; *C_i_*, intercellular CO_2_ concentration; *g_s_*, stomatal conductance; *SWC*, volumetric soil water content),total plant biomass, tuber weight, and tuber count during the sampling period. *P*-values are given for treatments and their interaction. Significant differences are given in bold (*P*≤0.05).

**Parameter**	***A***_***n***_	***C***_***i***_	***g***_***s***_	***SWC***	**Plant biomass**	**Tuber weight**	**Tuber count**
Temperature	0.070	**0.012**	0.127	0.863	0.978	0.359	0.400
Soil moisture	**0.020**	**0.010**	**0.001**	**0.002**	**0.008**	**0.023**	0.233
Temp.:moisture	0.522	0.110	0.174	0.845	0.565	0.892	0.486

**Table 2.** Biomass and tuber analyses after sampling period Total plant biomass (dry weight), tuber weight (fresh weight), and tuber count (number of potatoes) after the sampling period. Potato plants were treated with a combination of T_low_ (low temperature), T_high_ (high temperature), and wet or dry conditions. Means ±SE are given (*n*=3). Refer to Table 1 for statistical analysis.

	**Treatments**			
**Parameter**	**T**_**low**_ **wet**	**T**_**low**_ **dry**	**T**_**high**_ **wet**	**T**_**high**_ **dry**
Total biomass (g)	10.6±1.4	7.8±1.2	10.3±1	8.1±0.5
Tuber weight (g)	513.9±18.4	458.3±15	481.4±15.9	430±24.2
Tuber count (no.)	21.3±2.7	20.2±1.7	19.3±1.2	19±2.3

### Carbon isotopes in potato leaves

#### Daily cycles of δ^13^C_R_ and δ^13^C_leaf_

**Fig. 2.**
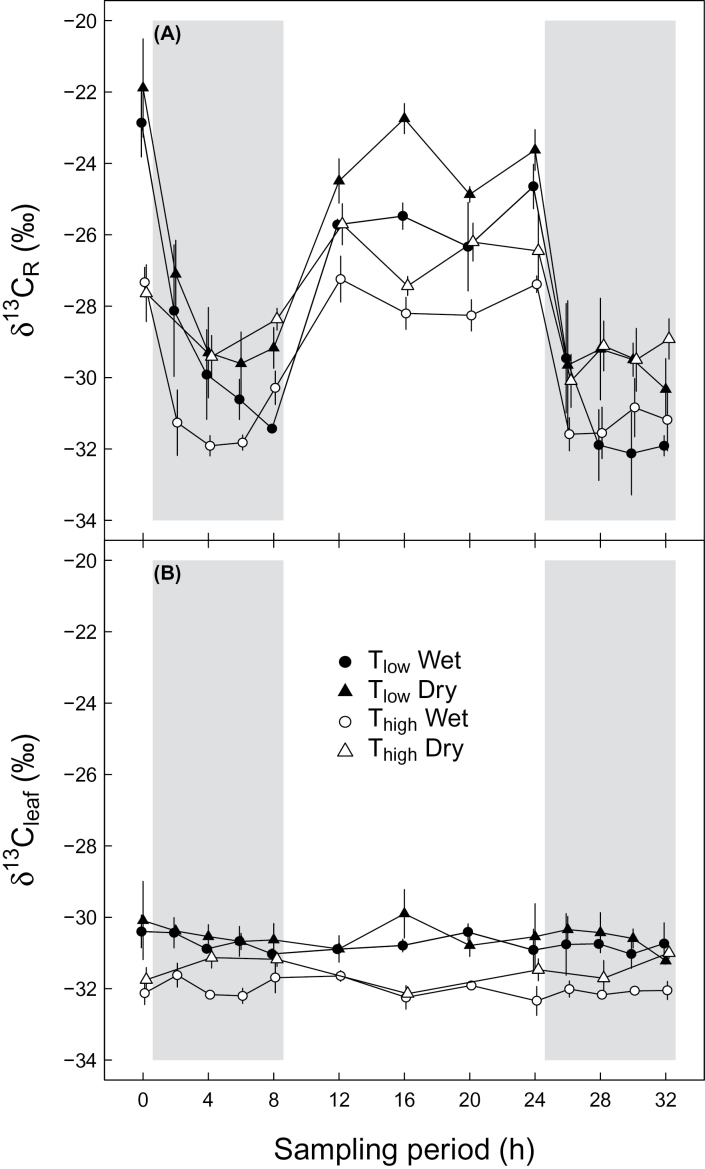
Daily cycles of the carbon isotopic composition of (A) leaf dark-respired CO_2_ (*δ* ^*13*^ *C*_*R*_) and (B) bulk leaves (*δ* ^*13*^ *C*_*leaf*_) under different environmental conditions during the sampling period. Potato plants were treated with a combination of T_low_ (low temperature; closed symbols), T_high_ (high temperature; open symbols), and wet (circles) or dry (triangles) conditions. Grey areas indicate nighttime. Means ±SE are given (*n*=3).

δ^13^C values of leaf dark-respired CO_2_ (*δ* ^*13*^ *C*_*R*_) varied significantly over time (*P*≤0.001, Table 3) with values in the range of −21.9‰ and −32‰, declining strongly during nighttime and increasing again during the daytime for all four treatments (Fig. 2A). An interaction between temperature and time showed that the influence of temperature differed with time (*P*=0.014, Table 3). Daytime *δ* ^*13*^ *C*_*R*_ values under T_high_ were up to 4.7‰ more negative compared to those under T_low_, independent of soil moisture conditions, whereas nighttime *δ* ^*13*^ *C*_*R*_ values of both temperature treatments were very similar, particularly in the second night. Dry soil moisture conditions caused less negative *δ* ^*13*^ *C*_*R*_ values compared to those under wet conditions during the daily cycle (*P*=0.013, Table 3), with a maximum difference of 2.7‰, independent of temperature treatments. On average, the difference between daytime and nighttime *δ* ^*13*^ *C*_*R*_ values was highest under T_low_ and wet conditions, at 5.7‰, and lowest under T_high_ and dry conditions, at 2.5‰.

**Table 3.** Environmental influences on leaf dark-respired CO_2_ and respiratory carbon sources Results of linear mixed effects models testing the effects of temperature (low, high) and soil moisture (wet, dry) on δ^13^C values in different putative leaf respiratory carbon sources, bulk leaves (*δ* ^*13*^ *C*_*leaf*_), and in leaf dark-respired CO_2_ (*δ* ^13^C_*R*_), as well as on concentrations of different carbon sources during the sampling period. Results for fructose are affected by co-elution with other compounds. *P*-values are given for treatments, time, and their interactions. Significant differences are given in bold (*P*≤0.05).

	δ^13^C							
Parameter	Fructose	Glucose	Sucrose	Malate	Citrate	Starch	*δ* ^*13*^ *C*_*Leaf*_	*δ* ^*13*^ *C*_*R*_
Temperature	**0.019**	**0.004**	**0.028**	**0.015**	n.a.	0.107	**0.022**	**0.044**
Soil moisture	**0.001**	**0.001**	**0.001**	**0.049**	**0.009**	**0.046**	**0.005**	**0.013**
Time	**0.001**	0.195	0.081	0.198	0.052	**0.001**	0.066	**0.001**
Temp.:moisture	**0.035**	0.063	0.543	**0.017**	n.a.	0.270	0.165	0.875
Temp.:time	0.256	**0.008**	**0.003**	0.807	n.a.	0.113	0.812	**0.014**
Moisture:time	0.061	0.291	**0.002**	0.060	0.411	**0.032**	0.596	0.883
	**Concentration**							
**Parameter**	**Fructose**	**Glucose**	**Sucrose**	**Malate**	**Citrate**	**Starch**		
Temperature	0.663	0.352	0.142	**0.011**	n.a.	**0.002**		
Soil moisture	**0.001**	**0.001**	**0.031**	0.999	0.052	**0.001**		
Time	**0.016**	0.927	**0.001**	**0.035**	0.110	**0.001**		
Temp:moisture	0.475	0.705	0.462	0.796	n.a.	**0.001**		
Temp.:time	0.901	0.847	0.113	0.387	n.a.	0.324		
Moisture:time	0.831	0.629	0.063	0.889	0.895	0.071		

n.a., not available

The bulk leaf material reflects all environmental conditions experienced during the whole growth period. *δ* ^*13*^ *C*_*leaf*_ of all treatments showed no changes during the sampling period and no interactions between treatments and time (Fig. 2B; Table 3). Under T_high_, *δ* ^*13*^ *C*_*leaf*_ values were up to 2.2‰ more negative compared to those under T_low_, resulting in a significant temperature effect independent of soil moisture conditions (*P*=0.022, Table 3). Similarly, soil moisture showed a significant effect on *δ* ^*13*^ *C*_*leaf*_ (*P*=0.005, Table 3), independent of temperature treatments, with values up to 1.1‰ less negative under dry than under wet conditions mainly during nighttime.

#### δ^13^ *C*_RS_ of soluble carbohydrates, organic acids, and starch

**Fig. 3.**
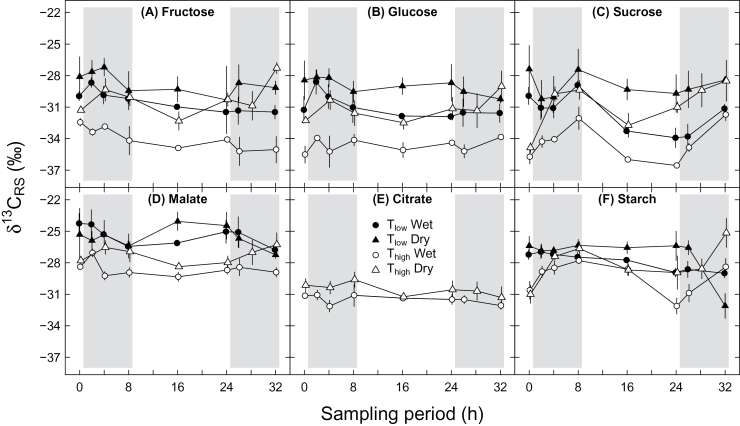
Daily cycles of the carbon isotopic composition of different leaf respiratory carbon sources (*δ* ^*13*^ *C*_*RS*_) under different environmental conditions during the sampling period: (A) fructose, (B) glucose, (C) sucrose, (D) malate, (E) citrate, and (F) starch. Potato plants were treated with a combination of T_low_ (low temperature; closed symbols), T_high_ (high temperature; open symbols), and wet (circles) or dry (triangles) conditions. Results for fructose are affected by co-elution with other compounds. Grey areas indicate nighttime. Means ± SE are given (*n*=2–3).

Highest δ^13^C values in putative leaf respiratory carbon sources (*δ* ^*13*^ *C*_*RS*_) were found in the organic acid malate, while soluble carbohydrates (fructose, glucose and sucrose) exhibited generally lowest *δ* ^*13*^ *C*_*RS*_ values (Fig. 3). *δ* ^*13*^ *C*_*RS*_ of soluble carbohydrates of all treatments were in the range of −27.2‰ and −36.6‰. More negative *δ* ^*13*^ *C*_*RS*_ values of glucose and sucrose under T_high_ compared to those under T_low_ were found, independent of soil moisture conditions, while less negative *δ* ^*13*^ *C*_*RS*_ values under dry conditions compared to those under wet conditions were observed, independent of temperature treatments (Fig. 3B, C; Table 3). Significant interactions between temperature and time for *δ* ^*13*^ *C*_*RS*_ of glucose (*P*=0.008, Table 3) and sucrose (*P*=0.003, Table 3) showed that daily cycles differed between temperatures. Additionally, soil moisture conditions caused significant temporal variations during the daily cycle in *δ* ^*13*^ *C*_*RS*_ of sucrose (*P*=0.002, Table 3).

**Fig. 4.**
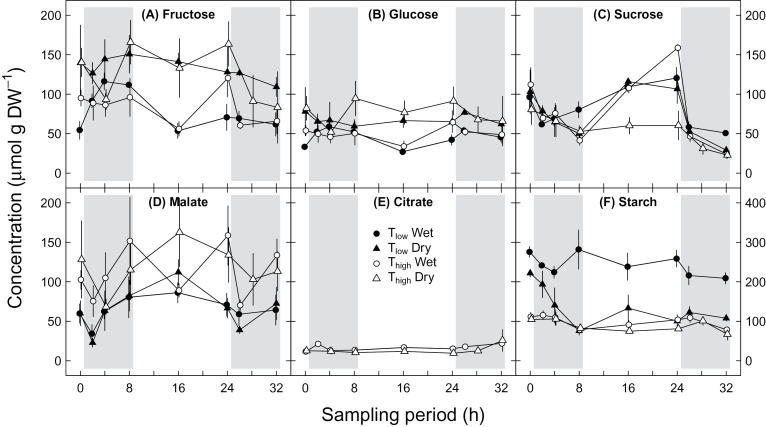
Daily cycles of the concentration of different leaf respiratory carbon sources under different environmental conditions during the sampling period: (A) fructose, (B) glucose, (C) sucrose, (D) malate, (E) citrate, and (F) starch. Potato plants were treated with a combination of T_low_ (low temperature; closed symbols), T_high_ (high temperature; open symbols), and wet (circles) or dry (triangles) conditions. Grey areas indicate nighttime. Results for fructose are affected by co-elution with other compounds. To facilitate comparison with other metabolites, sucrose concentrations were multiplied by 2 to count for hexose units, while starch concentrations are given in molarity of starch monomers. Note different y-axis scale in (F). Means ±SE are given (*n*=2–3).

We observed significant linear relationships between fructose and glucose for *δ* ^*13*^ *C*_*RS*_ (r^2^=0.74, *P*≤0.001) and concentration values (r^2^=0.8, *P*≤0.001), while relationships between the other *δ* ^*13*^ *C*_*RS*_ values and concentrations of different carbon sources were weaker (data not shown). However, the deviant results for *δ* ^*13*^ *C*_*RS*_ of fructose in comparison to the other sugars are assumed to reflect peak overlap issues of this sugar (Tables 3, 4). This is clearly reflected also in the concentration results (Fig. 4A). Consequently, the fructose results will not be discussed further in detail.

**Table 4.** Relationships between δ^13^C of leaf dark-respired CO_2_ and δ^13^C of respiratory carbon sources Linear regression analyses relating δ^13^C of leaf dark-respired CO_2_ to δ^13^C of putative respiratory carbon sources and to δ^13^C of bulk leaves (*δ* ^*13*^ *C*_*leaf*_) across all environmental conditions for daytime (0h, 16h, 24h), for nighttime (2h, 4h, 8h, 26h, 28h, 32h), and for the total daily cycle (sampling period over 32h). Results for fructose are affected by co-elution with other compounds. Generic regression equation *y*=*mx*+*b* was used. r^2^ values are given, stars indicate *P*-values. All correlation coefficients were positive.

	**r** ^**2**^		
**Putative carbon sources**	**Daytime**	**Nighttime**	**Daily**
Fructose	0.35***	0.34***	0.12***
Glucose	0.54***	0.34***	0.13***
Sucrose	0.59***	0.20***	0.04*
Malate	0.69***	0.36***	0.26***
Citrate	0.67***	0.28**	0.17**
Starch	0.48***	0.16**	0.06*
*δ* ^*13*^ *C*_*Leaf*_	0.63***	0.33***	0.20***

*, *P*≤0.05; **, *P*≤0.01; ***, *P*≤0.001

*δ* ^*13*^ *C*_*RS*_ of malate (Fig. 3D) in the range of −24‰ and −29.3‰ and *δ* ^*13*^ *C*_*RS*_ of citrate (Fig. 3E) in the range of −29.6‰ and −32.1‰ showed no temporal variations (*P*=0.198 and *P*=0.052 for malate and citrate, respectively, Table 3). Significant interactions between temperature and soil moisture treatments were observed for *δ* ^*13*^ *C*_*RS*_ of malate (*P*=0.017; Table 3), resulting in larger differences between *δ* ^*13*^ *C*_*RS*_ values of soil moisture conditions under T_high_ than under T_low_ (Fig. 3D). Citrate showed less negative *δ* ^*13*^ *C*_*RS*_ values under dry conditions than under wet conditions (*P*=0.009; Table 3).

*δ* ^*13*^ *C*_*RS*_ of starch of all treatments (Fig. 3F), ranging from −25.2‰ and −32.1‰, was influenced by soil moisture conditions (*P*=0.046, Table 3), independent of temperature treatments, while temperature showed no significant effect (*P*=0.107, Table 3). In addition, soil moisture conditions caused significant temporal variations during the daily cycle in *δ* ^*13*^ *C*_*RS*_ of starch (*P*=0.032, Table 3).

### Concentrations of soluble carbohydrates, organic acids, and starch

Concentrations of glucose of all treatments (Fig. 4B), ranging from 27 to 95 µmol g DW^-1^, showed no temporal variations (*P*=0.927, Table 3). In contrast, concentrations of sucrose (Fig. 4C) in the range of 23 to 159 µmol g DW^-1^ showed clear daily variations (*P*≤0.001, Table 3), with highest concentrations for all treatments by the end of the day, except for T_high_ and dry conditions. Glucose concentrations were significantly higher under dry than under wet conditions (*P*≤0.001, Table 3), while converse results were observed for sucrose (*P*=0.031, Table 3). Generally, no effect of temperature on the concentration of any soluble carbohydrate was observed.

Malate concentrations of all treatments (Fig. 4D), ranging from 23 to 163 µmol g DW^-1^, showed a daily pattern with declining concentrations in the beginning of the night and an increase after 2–4h in the dark (*P*=0.035, Table 3). In contrast to soluble carbohydrates, malate concentrations were significantly higher under T_high_ than under T_low_ (*P*=0.011, Table 3), but were not affected by soil moisture treatments (*P*=0.999, Table 3). Citrate concentrations under T_high_ of ~15 µmol g DW^-1^ were the lowest of all measured putative carbon sources available for leaf dark respiration and showed no changes due to soil moisture treatments and time (Fig. 4E; Table 3).

Starch concentrations (Fig. 4F), ranging from 67 to 282 µmol g DW^-1^, showed significant temporal variations (*P*≤0.001, Table 3), independent of any treatment. The average starch concentration of 243 µmol g DW^-1^ under T_low_ and wet conditions was clearly higher (~2.5 times) compared to those under other treatments. In addition, interactions between temperature and soil moisture treatments led to smaller differences between the values of wet and dry conditions under T_high_ compared to those under T_low_ (*P*≤ 0.001, Table 3).

### Linear relationships between *δ* ^*13*^ *C*_*R*_ and *δ* ^*13*^ *C*_*RS*_

Linear regression analyses were performed to understand the biochemical link between *δ* ^*13*^ *C*_*R*_ and *δ* ^*13*^ *C*_*RS*_ across all treatments (Table 4; Supplementary Fig. S1). *δ* ^*13*^ *C*_*RS*_ of malate explained most of the daily variation of *δ* ^*13*^ *C*_*R*_ (r^2^=0.26, *P*≤ 0.001), while the explanatory power of fructose, glucose, and citrate was lower. The lowest linear relationships during the daily cycle were found between *δ* ^*13*^ *C*_*R*_ and *δ* ^*13*^ *C*_*RS*_ of sucrose and starch. Due to the high daily variations in *δ* ^*13*^ *C*_*R*_ we carried out the same analysis separately for daytime and nighttime. Daytime linear relationships were generally stronger than during nighttime, with *δ* ^*13*^ *C*_*R*_ strongly related to *δ* ^*13*^ *C*_*RS*_ of malate, citrate, and *δ* ^*13*^ *C*_*leaf*_ (r^2^>0.6, *P*≤0.001), but lower related to *δ* ^*13*^ *C*_*RS*_ of soluble carbohydrates and starch. During nighttime, *δ* ^*13*^ *C*_*RS*_ of malate explained 36% of the variation in *δ* ^*13*^ *C*_*R*_, but *δ* ^*13*^ *C*_*RS*_ of fructose and glucose, as well as *δ* ^*13*^ *C*_*leaf*_, showed similarly high explanatory power.

### Influence of environmental drivers and carbon sources on *δ* ^*13*^ *C*_*R*_

Furthermore, a stepwise (backward) multiple linear regression analysis was performed to identify environmental drivers and carbon sources influencing *δ* ^*13*^ *C*_*R*_ (Table 5). Daytime/nighttime showed the strongest positive effect on *δ* ^*13*^ *C*_*R*_ (β=0.73, *P*≤0.001), while *δ* ^*13*^ *C*_*RS*_ of malate was the carbon source that affected *δ* ^*13*^ *C*_*R*_ most (β=0.4, *P*≤0.001). By comparison, the influence of *δ* ^*13*^ *C*_*RS*_ of starch and soil moisture conditions on *δ* ^*13*^ *C*_*R*_ values was minor.

**Table 5.** Environmental drivers and carbon sources influencing δ^13^C of leaf dark-respired CO_2_ Result of stepwise (backward) multiple linear regression analysis showing the best-fit combination of independent environmental drivers (temperature, soil moisture, daytime/nighttime), time, and δ^13^C of glucose, sucrose, malate, and starch as variables influencing δ^13^C of leaf dark-respired CO_2_ (*δ* ^*13*^ *C*_*R*_) during the sampling period in potato leaves. Standardized β-coefficients and *P*-values are given.

Drivers and carbon sources influencing δ*^13^C_R_*	Standardized β-coefficient	*P*-value
Daytime/nighttime	0.73	<0.001
Malate	0.40	<0.001
Starch	0.11	0.019
Soil moisture	0.14	0.013

## Discussion

This study clearly demonstrates that different temperature and soil moisture conditions influence δ^13^C of leaf dark-respired CO_2_ (*δ* ^*13*^ *C*_*R*_), δ^13^C of different putative leaf respiratory carbon sources (*δ* ^*13*^ *C*_*RS*_), and concentrations of carbon sources during a daily cycle in potato leaves. Furthermore, our findings strongly indicate malate as a key carbon source of daytime and nighttime *δ* ^*13*^ *C*_*R*_ across different environmental conditions.

### Influence of temperature and soil moisture on isotopic compositions

After 2 weeks of treatment, we already found a clear temperature effect on *δ* ^*13*^ *C*_*leaf*_, with up to 2.2‰ more negative *δ* ^*13*^ *C*_*leaf*_ values under T_high_ conditions compared to those under T_low_ conditions (Fig. 2B). This is in agreement with a study showing more negative δ^13^C values with increasing temperature for bulk leaves of *Xanthium* species (Smith *et al.*, 1976). Similar to Tcherkez *et al.* (2003) under short-term temperature treatments, we observed more negative *δ* ^*13*^ *C*_*R*_ value with increasing temperature (Fig. 2A), but due to our long-term treatment we found also more negative *δ* ^*13*^ *C*_*RS*_ values (Fig. 3). On the other hand, dry conditions in both of the temperature treatments caused less negative *δ* ^*13*^ *C*_*leaf*_, *δ* ^*13*^ *C*_*R*_, and *δ* ^*13*^ *C*_*RS*_ values compared to those under wet conditions, which is consistent with previous studies under controlled conditions (Duranceau *et al.*, 1999; Ghashghaie *et al.*, 2001).

The isotopic results under the different environmental conditions can be directly linked to the leaf gas exchange observed during the 32h sampling period (day 15 of the treatment period). Increasing temperature caused lower *A*_*n*_ values under both soil moisture conditions (Fig. 1A; Table 1), indicating that plants under T_high_ were beyond the photosynthetic optimum. This result is in agreement with earlier studies, showing that cold-adapted potato plants have reduced rates of photosynthesis with temperatures above 20°C (Levy and Veilleux, 2007). Additionally, *A*_*n*_ might be also influenced by leaf ageing, since *A*_*n*_ decreased under all treatments during the treatment period. On the other hand, *g*_*s*_ tended to higher values with increasing temperature, but only under wet conditions (Fig. 1C; Table 1). An increase of *g*_*s*_ under T_high_ might be triggered by increasing transpiration rates, which could be a physiological response to compensate reduced rates of *A*_*n*_ by cooling the leaf temperature under T_high_ conditions. However, this was only observed in plants under T_high_ and well-watered conditions, when *SWC* was high. Subsequently, lower carbon fixation and higher CO_2_ diffusion into the stomatal cavities under T_high_, in comparison to T_low_, caused an increase of *C*_*i*_ (Fig. 1B) and more negative *δ* ^*13*^ *C*_*R*_ and *δ* ^*13*^ *C*_*RS*_ values (Table 6). Furthermore, dry soil moisture conditions caused reduced rates of *A*_*n*_ and *g*_*s*_ compared to those under wet conditions (Fig. 1A, C; Table 1), independent of temperature treatments. This can be explained with the severe drought stress, reflecting low *SWC* values (Fig. 1D). Consequently, plants under dry conditions experienced reduced CO_2_ diffusion into the stomatal cavities, leading to lower *C*_*i*_ and less negative δ^13^C values (Table 6).

**Table 6.** Coherence between leaf physiological parameters and δ^13^C values. Leaf physiological parameters and δ^13^C values during the sampling period in potato plants under different treatments compared to those in potato plants growing under T_low_ and wet conditions The following variables were considered: *A*_*n*_, net assimilation rate; *C*_*i*_, intercellular CO_2_ concentration; *g*_*s*_, stomatal conductance; *δ* ^*13*^ *C*_*R*_, *δ* ^*13*^ *C* of leaf dark-respired CO_2_; *δ* ^*13*^ *C*_*RS*_, δ^13^C of different putative respiratory carbon sources (fructose, glucose, sucrose, starch, and malate). Arrows indicate strong (↑, ↓), intermediate (↗, ↘), or no changes (→) due to the influence of treatment combinations (T_low_, low temperature; T_high_, high temperature; and wet or dry conditions).

Treatments	*A*_*n*_	*g*_*s*_	*C*_*i*_	δ^13^C_R_	δ^13^C_RS_
T_low_ dry	↘	↓	↓	↑	↑
T_high_ wet	↘	↑	↑	↓	↓
T_high_ dry	↓	↓	↗	→	→

Plants under T_high_ and dry conditions showed the lowest performance during the sampling period compared to plants under other treatments, which is reflected in low *A*_*n*_ values (Fig. 1A), plant biomass, tuber weight and tuber count (Table 2). *δ* ^*13*^ *C*_*R*_ and *δ* ^*13*^ *C*_*RS*_ in these plants were expected to be the most positive compared to other treatments due to a severe drought caused by the double effect of high temperature and dry soil moisture. Instead, *δ* ^*13*^ *C*_*R*_ and *δ* ^*13*^ *C*_*RS*_ of the plants under the highest stress level (T_high_ and dry conditions) were rather similar to those under lowest stress level (T_low_ and wet conditions). This was particularly observed for *δ* ^*13*^ *C*_*RS*_ of soluble carbohydrates and starch (Fig. 3). Again, this is an indicator of low *A*_*n*_ under T_high_ and dry conditions, resulting in a moderate reduction of *C*_*i*_, while at the same time *g*_*s*_ strongly reduces CO_2_ diffusion into the stomatal cavities, causing an increase of *C*_*i*_. Consequently, this led to intermediate *δ* ^*13*^ *C*_*R*_ and *δ* ^*13*^ *C*_*RS*_ values under T_high_ and dry conditions (Table 6). In summary our findings indicate that combined effects of temperature and soil moisture conditions on *δ* ^*13*^ *C*_*R*_ and *δ* ^*13*^ *C*_*RS*_ could cancel out the individual effect of each driver.

### Environmental influences on concentrations of putative carbon sources

Soil moisture and temperature affected concentrations of putative leaf respiratory carbon sources differently. Sucrose concentration decreased under dry conditions (Fig. 4C; Table 3), which is in contrast to the recent study by Lemoine *et al.* (2013). This may be explained by reduced rates of sucrose synthesis due to lowering of the sucrose phosphate synthase reaction (SPS) (Vu *et al.*, 1998). The decrease in the enzyme activity is probably triggered by limited rates of phloem sugar transport observed under drought (Ruehr *et al.*, 2009). This in turn could be an explanation for lower plant biomass and tuber weight/count in response to higher temperatures and dry conditions (Tables 1, 2). Subsequently, the increase of fructose and glucose concentrations under drought may also be a consequence of lower SPS activity (Fig. 4A, B; Table 3), since the demand for both hexoses for sucrose synthesis was reduced. Additionally, increasing fructose and glucose concentrations under drought might have osmotic functionality, maintaining metabolic activity (Lemoine *et al.*, 2013).

On the other hand, malate concentrations increased with temperature (Fig. 4D; Table 3), which is most likely a consequence of higher PEPC activity (Chinthapalli *et al.*, 2003). Higher malate concentrations may also support respiratory processes in the KC or regulation of stomatal opening (Finkemeier and Sweetlove, 2009). Moreover, decreased starch concentrations in leaves under treatments with higher environmental stress than T_low_ and wet conditions (Fig. 4F) were similar to previous findings (Lemoine *et al.*, 2013). The result also supports the assumption that reduced amounts of assimilated carbon due to lower *A*_*n*_ under T_high_ or dry conditions were used for maintenance of biochemical processes rather than for carbon storage. Additionally, this indicates that plants under T_high_ or dry conditions were under severe environmental stress.

### Malate as a key respiratory carbon source of daytime and nighttime *δ* ^*13*^ *C*_*R*_

The daily cycle of *δ* ^*13*^ *C*_*R*_ was highly variable, showing less negative daytime and more negative nighttime values, while *δ* ^*13*^ *C*_*RS*_ values generally showed lower changes during the same period (Figs 2A, 3; Table 3). *δ* ^*13*^ *C*_*RS*_ values of all treatments compared to *δ* ^*13*^ *C*_*R*_ values were more negative for soluble carbohydrates (up to 9.3‰) and citrate (up to 4.1‰), but also less negative for starch (up to 4‰) and malate (up to 5.2‰) during the daily cycle (Figs 2A, 3). In particular, malate was strongly enriched in ^13^C, by up to 8.8‰, compared to all other putative carbon sources (Fig. 3). This was similar to a previous study investigating metabolites in potato leaves (Gleixner *et al.*, 1998) and indicates a possible biochemical link between ^13^C enriched leaf dark-respired CO_2_ and ^13^C enriched malate.

For a better understanding of the overall biochemical connections between *δ* ^*13*^ *C*_*R*_ and different putative carbon sources, we carried out linear regression analyses, independent of environmental conditions (Table 4; Supplementary Fig. S1). The daily linear relationship between *δ* ^*13*^ *C*_*R*_ and *δ* ^*13*^ *C*_*RS*_ of malate was stronger compared to all other putative carbon sources (r^2^=0.26, *P*≤0.001). The strength of this relationship increased for *δ* ^*13*^ *C*_*R*_ and *δ* ^*13*^ *C*_*RS*_ of malate when considering daytime (r^2^=0.69, *P*≤0.001) and nighttime (r^2^=0.36, *P*≤0.001) separately. Moreover, relationships of *δ* ^*13*^ *C*_*R*_ with *δ* ^*13*^ *C*_*RS*_ of malate were stronger than those of *δ* ^*13*^ *C*_*R*_ with δ^13^C of bulk leaves (reflects the average δ^13^C value of all respiratory substrates), which was, however, not the case for most relationships of *δ* ^*13*^ *C*_*R*_ with other carbon sources.

Please note that comparisons between daytime and nighttime relationships must be done carefully (Table 4) due to the bias caused by LEDR in daytime *δ* ^*13*^ *C*_*R*_, which depends on the amount of assimilated carbon (Priault *et al.*, 2009) and probably also on environmental conditions. LEDR is considered to be fuelled by malate (Atkin *et al.*, 1998; Barbour *et al.*, 2007; Gessler *et al.*, 2009; Werner and Gessler, 2011). Consequently, the strong daytime relationship between *δ* ^*13*^ *C*_*R*_ and *δ* ^*13*^ *C*_*RS*_ of malate might be explained by a higher respiratory consumption of malate during the LEDR period, provoking less negative daytime *δ* ^*13*^ *C*_*R*_ values (Fig. 2A). Furthermore, transferring light-acclimated leaves into darkness is suggested to lead to reassembly of the KC by activation of light-inhibited enzymatic reactions of the cycle (Tcherkez *et al.*, 2005; Sweetlove *et al.*, 2010; Werner and Gessler, 2011). During LEDR the KC might not be fully active, leading to changes in metabolic fluxes and isotope fractionations, which may not occur during nighttime when KC is fully reassembled (Werner *et al.*, 2011). This could be an important factor, explaining light-dark differences in the relationships between *δ* ^*13*^ *C*_*R*_ and *δ* ^*13*^ *C*_*RS*_ of different carbon sources in this study (Table 4).

In contrast to malate, *δ* ^*13*^ *C*_*RS*_ of carbon storage compounds, such as starch and sucrose, were less related to *δ* ^*13*^ *C*_*R*_ during daytime and nighttime (Table 4). This can particularly be explained for starch due to the fact that its isotopic composition is always a mix of fresh and old assimilates, constraining good relationships with the isotopic composition of recently respired CO_2_. Moreover, the high daytime relationship between *δ* ^*13*^ *C*_*R*_ and *δ* ^*13*^ *C*_*RS*_ of citrate might be explained by the close biochemical relationship of citrate with malate via the mitochondrial malate dehydrogenase and citrate synthase (Voet and Voet, 2011). However, citrate was ^13^C depleted and showed very low concentrations compared to other carbon sources (Figs 3E, 4E), contradicting the role of citrate as an important carbon source of *δ* ^*13*^ *C*_*R.*_

We also observed regular decreases in malate concentrations in the beginning of the night across all environmental conditions (Fig. 4D), as observed in previous studies (Urbanczyk-Wochniak *et al.*, 2005; Gessler *et al.*, 2009), which may reflect the use of malate for respiratory processes shortly upon darkening, e.g. LEDR. It has also been suggested that malate accumulates during daytime (Barbour *et al.*, 2007; Gessler *et al.*, 2009; Werner and Gessler, 2011). However, low temporal variations in malate concentrations during daytime do not support this hypothesis.

Furthermore, the hypothesis that *δ* ^*13*^ *C*_*R*_ is influenced by the putative carbon source malate across all treatments was also indicated by a stepwise multiple linear regression analysis (Table 5, *P*-values). The findings are in line with our other observations showing that (i) daytime and nighttime periods have a clear influence on *δ* ^*13*^ *C*_*R*_ (Fig. 2A); (ii) *δ* ^*13*^ *C*_*RS*_ of malate has the strongest influence on *δ* ^*13*^ *C*_*R*_ compared to all other putative carbon sources; and (iii) influences of other environmental drivers and carbon sources are weaker and less significant compared to daytime/nighttime and malate. Overall, the findings strongly indicate *δ* ^*13*^ *C*_*RS*_ of malate as a key carbon source of *δ* ^*13*^ *C*_*R*_ during the daily cycle across all environmental conditions within this study.

**Fig. 5.**
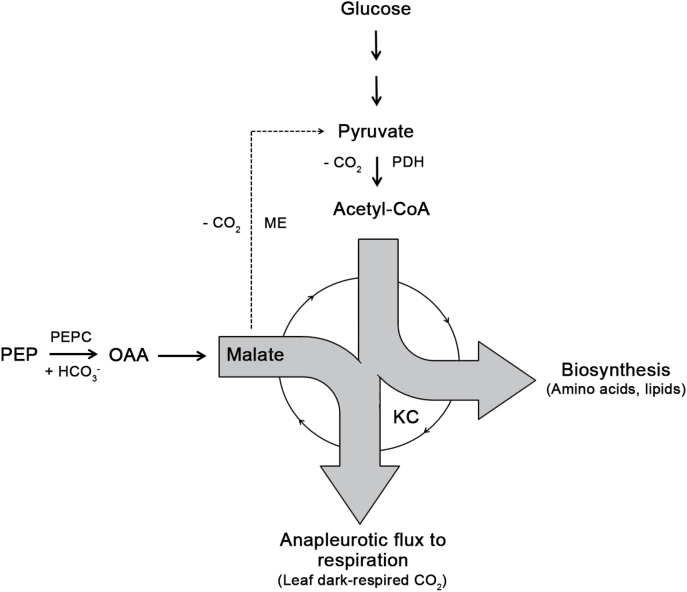
Simplified schematic of respiratory processes in potato leaves. Breakdown of glucose during glycolysis produces pyruvate, which is converted into CO_2_ and acetyl-CoA by the pyruvate dehydrogenase reaction (PDH). Acetyl-CoA is used for biosynthesis rather than for respiration, causing a drain of Krebs cycle (KC) intermediates. An anapleurotic flux via phosphoenolpyruvate carboxylase (PEPC) replenishes KC intermediates to maintain the functionality of the KC. Malate enters the KC and is used as carbon source for leaf dark-respired CO_2_. Dashed line indicates an alternative CO_2_ producing reaction via malic enzyme (ME). PEP, phosphoenolpyruvate; OAA, oxaloacetate.

A mechanistic explanation for the respiratory use of malate can be found within the amphibole functionality of the KC and associated reactions (malic enzyme, PDH; Fig. 5). Generally, the breakdown of glucose during glycolysis produces pyruvate. Leaf feeding experiments using position-specific ^13^C labelled pyruvate have shown in different species that respiration of the C-1 position of pyruvate is higher compared to respiration of the C-2 and C-3 position of pyruvate during daytime (Priault *et al.*, 2009; Wegener *et al.*, 2010), as well as during nighttime (Werner *et al.*, 2009). This clearly indicates that acetyl-CoA (C-2 and C-3 position of pyruvate) from the PDH reaction, which enters the KC, is used for biosynthesis of diverse metabolic compounds (e.g. amino acids or lipids), rather than for respiration (Fig. 5). If this is true, withdrawn KC intermediates must be refilled due to stoichiometric reasons to maintain the functionality of the KC. This could be achieved by an anapleurotic flux via PEPC, which has often been described as replenishing KC intermediates (Melzer and O’Leary, 1987; Savidge and Blair, 2004). The PEPC reaction produces ^13^C-enriched oxaloacetate, of which the greatest proportion is directly converted into malate via the malate dehydrogenase reaction. A breakdown of this malate pool within the KC or associated reactions (malic enzyme, PDH) would then produce ^13^C-enriched leaf dark-respired CO_2_ (Fig. 5), explaining the close relationship between *δ* ^*13*^ *C*_*R*_ and *δ* ^*13*^ *C*_*RS*_ of malate found in this study. Moreover, malate is supposed to be ^13^C enriched at the C-4 position via PEPC, while other positions of the molecule are ^13^C depleted via glycolysis (Melzer and O’Leary, 1987; Savidge and Blair, 2004), causing dampening of the ^13^C enrichment at the C-4 position when measuring δ^13^C of the whole malate molecule (Fig. 3D). Therefore, slight changes in δ^13^C of malate may indicate higher changes at the C-4 position, which can be decarboxylated by the malic enzyme reaction or within the KC and thus be highly relevant for variations in *δ* ^*13*^ *C*_*R*_. In brief, our findings strongly suggest that *δ* ^*13*^ *C*_*RS*_ of malate has a strong influence on *δ* ^*13*^ *C*_*R*_ during daytime, as well as nighttime, across different environmental conditions in this study and that their biochemical link is driven by an anapleurotic flux via PEPC, replenishing KC intermediates.

## Conclusions

Here we showed for the first time results of δ^13^C of leaf dark-respired CO_2_ and δ^13^C of putative respiratory carbon sources under the combined influence of controlled temperature and soil moisture conditions on a daily basis in a C_3_ plant. Overall, we found that *δ* ^*13*^ *C*_*R*_ values generally reflect changes in *δ* ^*13*^ *C*_*RS*_ values in putative respiratory carbon sources due to the influence of different temperature and soil moisture treatments on leaf physiological parameters. It is worth noting that the temperature in this study exceeded the photosynthetic optimum of the potato plants under T_high_, unexpectedly leading to more negative δ^13^C values under T_high_ and dry conditions than those observed under T_low_ and dry conditions. This demonstrates that conclusions about the individual influence of an environmental driver on δ^13^C values should be drawn carefully and that verification of the isotopic results by gas exchange measurements is mandatory. Moreover, our findings indicate malate as a key respiratory carbon source of leaf dark-respired CO_2_ in potato plants. This could also be the case in plant species comparable with potato, but should not be generalized and transferred to respiratory processes in species of different functional groups such as trees or shrubs without verification. Please note that for exact quantification of the respiratory contribution of malate in comparison to other metabolites more knowledge about metabolic fluxes and turnover rates is necessary. Finally, for subsequent studies on this topic we recommend the inclusion of isotopic measurements of malate or of the organic acid pool, given the strong indications observed herein for a biochemical link between δ^13^C of malate and δ^13^C of leaf dark-respired CO_2_.

## Supplementary data

Supplementary data are available at *JXB* online.

Supplementary Figure S1. Linear regressions between δ^13^C of leaf dark-respired CO_2_ (*δ* ^*13*^ *C*_*R*_) and δ^13^C of different putative respiratory carbon sources (*δ* ^*13*^ *C*_*RS*_) across all environmental conditions for daytime, for nighttime, and for the total daily cycle.

Supplementary Data

## Abbreviations:

*δ*^*13*^*C*_*R*_: carbon isotopic composition of leaf dark-respired CO_2_
*δ*^*13*^*C*_*RS*_: carbon isotopic composition of putative leaf respiratory carbon sources
*A*_*n*_: net assimilation rate
*C*_*i*_: intercellular CO_2_ concentration
CSIA: compound-specific isotope analysis
*g*_*s*_: stomatal conductance
HPLC: high performance liquid chromatography
KC: Krebs cycle
LEDR: light-enhanced dark respiration
ME: malic enzyme
OAA: oxaloacetate
PDH: pyruvate dehydrogenase
PEPC: phosphoenolpyruvate carboxylase
SPS: sucrose phosphate synthase
SWC: volumetric soil water content.

## References

[CIT0001] Atkin OK Evans JR Siebke K 1998 Relationship between the inhibition of leaf respiration by light and enhancement of leaf dark respiration following light treatment. Australian Journal of Plant Physiology 25, 437–443.

[CIT0002] Barbour MM Hunt JE Kodama N Laubach J McSeveny TM Rogers GN Tcherkez G Wingate L 2011 Rapid changes in δ^13^C of ecosystem-respired CO_2_ after sunset are consistent with transient 13C enrichment of leaf respired CO_2_ . New Phytologist 190, 990–1002. 21294737 10.1111/j.1469-8137.2010.03635.x

[CIT0003] Barbour MM McDowell NG Tcherkez G Bickford CP Hanson DT 2007 A new measurement technique reveals rapid post-illumination changes in the carbon isotope composition of leaf-respired CO_2_ . Plant, Cell & Environment 30, 469–482. 10.1111/j.1365-3040.2007.01634.x 17324233

[CIT0004] Boschker HTS Moerdijk–Poortvliet TCW van Breugel P Houtekamer M Middelburg JJ 2008 A versatile method for stable carbon isotope analysis of carbohydrates by high-performance liquid chromatography/isotope ratio mass spectrometry. Rapid Communications in Mass Spectrometry 22, 3902–3908. 18980267 10.1002/rcm.3804

[CIT0005] Bowling DR Pataki DE Randerson JT 2008 Carbon isotopes in terrestrial ecosystem pools and CO_2_ fluxes. New Phytologist 178, 24–40. 18179603 10.1111/j.1469-8137.2007.02342.x

[CIT0006] Chinthapalli B Murmu J Raghavendra AS 2003 Dramatic difference in the responses of phosphoenolpyruvate carboxylase to temperature in leaves of C_3_ and C_4_ plants. Journal of Experimental Botany 54, 707–714. 12554714 10.1093/jxb/erg078

[CIT0007] Coplen TB 2011 Guidelines and recommended terms for expression of stable-isotope-ratio and gas-ratio measurement results. Rapid Communications in Mass Spectrometry 25, 2538–2560. 21910288 10.1002/rcm.5129

[CIT0008] Craig H 1957 Isotopic standards for carbon and oxygen and correction factors for mass-spectrometric analysis of carbon dioxide. Geochimica et Cosmochimica Acta 12, 133–149.

[CIT0009] Critchley JH Zeeman SC Takaha T Smith AM Smith SM 2001 A critical role for disproportionating enzyme in starch breakdown is revealed by a knock-out mutation in *Arabidopsis* . The Plant Journal 26, 89–100. 11359613 10.1046/j.1365-313x.2001.01012.x

[CIT0010] Dubbert M Rascher KG Werner C 2012 Species-specific differences in temporal and spatial variation in δ^13^C of plant carbon pools and dark-respired CO_2_ under changing environmental conditions. Photosynthesis Research 113, 297–309. 22618996 10.1007/s11120-012-9748-3

[CIT0011] Duranceau M Ghashghaie J Badeck F Deleens E Cornic G 1999 δ^13^C of CO_2_ respired in the dark in relation to δ^13^C of leaf carbohydrates in *Phaseolus vulgaris* L. under progressive drought. Plant, Cell & Environment 22, 515–523.

[CIT0012] Farquhar GD Ehleringer JR Hubick KT 1989 Carbon isotope discrimination and photosynthesis. Annual Review of Plant Physiology and Plant Molecular Biology 40, 503–537.

[CIT0013] Finkemeier I Sweetlove LJ 2009 The role of malate in plant homeostasis. F1000 Biology Reports doi: 10.3410/B1-47. 10.3410/B1-47 PMC2924691 20948638

[CIT0014] Gessler A Tcherkez G Karyanto O Keitel C Ferrio JP Ghashghaie J Kreuzwieser J Farquhar GD 2009 On the metabolic origin of the carbon isotope composition of CO_2_ evolved from darkened light-acclimated leaves in *Ricinus communis* . New Phytologist 181, 374–386. 19121034 10.1111/j.1469-8137.2008.02672.x

[CIT0015] Ghashghaie J Badeck FW 2014 Opposite carbon isotope discrimination during dark respiration in leaves versus roots—a review. New Phytologist 201, 751–769. 24251924 10.1111/nph.12563

[CIT0016] Ghashghaie J Badeck FW Lanigan G Nogues S Tcherkez G Deleens E Cornic G Griffiths H 2003 Carbon isotope fractionation during dark respiration and photorespiration in C_3_ plants. Phytochemistry Reviews 2, 145–161.

[CIT0017] Ghashghaie J Duranceau M Badeck FW Cornic G Adeline MT Deleens E 2001 δ^13^C of CO_2_ respired in the dark in relation to d13C of leaf metabolites: comparison between *Nicotiana sylvestris* and *Helianthus annuus* under drought. Plant, Cell & Environment 24, 505–515.

[CIT0018] Gleixner G Schmidt H-L 1997 Carbon isotope effects on the fructose-1,6-bisphosphate aldolase reaction, origin for non-statistical ^13^C distributions in carbohydrates. Journal of Biological Chemistry 272, 5382–5387. 9038136 10.1074/jbc.272.9.5382

[CIT0019] Gleixner G Scrimgeour C Schmidt H-L Viola R 1998 Stable isotope distribution in the major metabolites of source and sink organs of *Solanum tuberosum* L.: a powerful tool in the study of metabolic partitioning in intact plants. Planta 207, 241–245.

[CIT0020] Goettlicher S Knohl A Wanek W Buchmann N Richter A 2006 Short-term changes in carbon isotope composition of soluble carbohydrates and starch: from canopy leaves to the root system. Rapid Communications in Mass Spectrometry 20, 653–660. 16444688 10.1002/rcm.2352

[CIT0021] Hettmann E Gleixner G Juchelka D 2005 IRM-LC/MS: δ^13^C analysis of organic acids in plants. Application Note 30075, Thermo Fisher Scientific.

[CIT0022] Hoch G Popp M Koerner C 2002 Altitudinal increase of mobile carbon pools in *Pinus cembra* suggests sink limitation of growth at the Swiss treeline. Oikos 98, 361–374.

[CIT0023] Hopkins WG 2006 Photosynthesis and Respiration . New York: Chelsea House, 88–109.

[CIT0024] Hymus GJ Maseyk K Valentini R Yakir D 2005 Large daily variation in ^13^C-enrichment of leaf-respired CO_2_ in two *Quercus* forest canopies. New Phytologist 167, 377–384. 15998391 10.1111/j.1469-8137.2005.01475.x

[CIT0025] Krummen M Hilkert AW Juchelka D Duhr A Schluter HJ Pesch R 2004 A new concept for isotope ratio monitoring liquid chromatography/mass spectrometry. Rapid Communications in Mass Spectrometry 18, 2260–2266. 15384146 10.1002/rcm.1620

[CIT0026] Lemoine R La Camera S Atanassova R 2013 Source-to-sink transport of sugar and regulation by environmental factors. Frontiers in Plant Science 4 doi: 10.3389/fpls.2013.00272. 10.3389/fpls.2013.00272 PMC3721551 23898339

[CIT0027] Levy D Veilleux RE 2007 Adaptation of potato to high temperatures and salinity—a review. American Journal of Potato Research 84, 487–506.

[CIT0028] Melzer E O’Leary MH 1987 Anapleurotic CO_2_ fixation by phosphoenolpyruvate carboxylase in C_3_ plants. Plant Physiology 84, 58–60. 16665405 10.1104/pp.84.1.58 PMC1056527

[CIT0029] Prater JL Mortazavi B Chanton JP 2006 Diurnal variation of the δ^13^C of pine needle respired CO_2_ evolved in darkness. Plant, Cell & Environment 29, 202–211. 10.1111/j.1365-3040.2005.01413.x 17080636

[CIT0030] Priault P Wegener F Werner C 2009 Pronounced differences in diurnal variation of carbon isotope composition of leaf respired CO_2_ among functional groups. New Phytologist 181, 400–412. 19121035 10.1111/j.1469-8137.2008.02665.x

[CIT0031] R Core Team. 2013 R: a language and environment for statistical computing. R Foundation for Statistical Computing, Vienna, Austria.

[CIT0032] Richter A Wanek W Werner RA 2009 Preparation of starch and soluble sugars of plant material for the analysis of carbon isotope composition: a comparison of methods. Rapid Communications in Mass Spectrometry 23, 2476–2488. 19603463 10.1002/rcm.4088

[CIT0033] Rinne KT Saurer M Streit K Siegwolf RTW 2012 Evaluation of a liquid chromatography method for compound-specific δ^13^C analysis of plant carbohydrates in alkaline media. Rapid Communications in Mass Spectrometry 26, 2173–2185. 22886814 10.1002/rcm.6334

[CIT0034] Rossmann A Butzenlechner M Schmidt H-L 1991 Evidence for a nonstatistical carbon isotope distribution in natural glucose. Plant Physiology 96, 609–614. 16668229 10.1104/pp.96.2.609 PMC1080814

[CIT0035] Ruehr NK Offermann CA Gessler A Winkler JB Ferrio JP Buchmann N Barnard RL 2009 Drought effects on allocation of recent carbon: from beech leaves to soil CO_2_ efflux. New Phytologist 184, 950–961. 19843305 10.1111/j.1469-8137.2009.03044.x

[CIT0036] Savidge WB Blair NE 2004 Patterns of intramolecular carbon isotopic heterogeneity within amino acids of autotrophs and heterotrophs. Oecologia 139, 178–189. 14986094 10.1007/s00442-004-1500-z

[CIT0037] Smith BN Oliver J Millan CM 1976 Influence of carbon source, oxygen concentration, light intensity, and temperature on ^13^C/^12^C ratios in plant tissues. Botanical Gazette 137, 99–104.

[CIT0038] Streit K Rinne KT Hagedorn F Dawes MA Saurer M Hoch G Werner RA Buchmann N Siegwolf RTW 2013 Tracing fresh assimilates through *Larix decidua* exposed to elevated CO_2_ and soil warming at the alpine treeline using compound-specific stable isotope analysis. New Phytologist 197, 838–849. 23252478 10.1111/nph.12074

[CIT0039] Sun W Resco V Williams DG 2009 Diurnal and seasonal variation in the carbon isotope composition of leaf dark–respired CO_2_ in velvet mesquite (*Prosopis velutina*). Plant, Cell & Environment 32, 1390–1400. 10.1111/j.1365-3040.2009.02006.x 19558412

[CIT0040] Sweetlove LJ Beard KFM Nunes-Nesi A Fernie AR Ratcliffe RG 2010 Not just a circle: flux modes in the plant TCA cycle. Trends in Plant Science 15, 462–470. 20554469 10.1016/j.tplants.2010.05.006

[CIT0041] Tcherkez G Cornic G Bligny R Gout E Ghashghaie J 2005 In vivo respiratory metabolism of illuminated leaves. Plant Physiology 138, 1596–1606. 15980193 10.1104/pp.105.062141 PMC1176429

[CIT0042] Tcherkez G Farquhar G Badeck F Ghashghaie J 2004 Theoretical considerations about carbon isotope distribution in glucose of C_3_ plants. Functional Plant Biology 31, 857–877. 10.1071/FP04053 32688955

[CIT0043] Tcherkez G Nogues S Bleton J Cornic G Badeck F Ghashghaie J 2003 Metabolic origin of carbon isotope composition of leaf dark-respired CO_2_ in French bean. Plant Physiology 131, 237–244. 12529531 10.1104/pp.013078 PMC166803

[CIT0044] Unger S Maguas C Pereira JS Aires LM David TS Werner C 2010 Disentangling drought-induced variation in ecosystem and soil respiration using stable carbon isotopes. Oecologia 163, 1043–1057. 20217141 10.1007/s00442-010-1576-6

[CIT0045] Urbanczyk-Wochniak E Baxter C Kolbe A Kopka J Sweetlove LJ Fernie AR 2005 Profiling of diurnal patterns of metabolite and transcript abundance in potato (*Solanum tuberosum*) leaves. Planta 221, 891–903. 15744496 10.1007/s00425-005-1483-y

[CIT0046] Usadel B Blasing OE Gibon Y Retzlaff K Hoehne M Gunther M Stitt M 2008 Global transcript levels respond to small changes of the carbon status during progressive exhaustion of carbohydrates in *Arabidopsis* rosettes. Plant Physiology 146, 1834–1861. 18305208 10.1104/pp.107.115592 PMC2287354

[CIT0047] Voet D Voet JG 2011 Biochemistry, 4th edition New York: Wiley, 789–822.

[CIT0048] Vu JCV Baker JT Pennanen AH Allen LH Bowes G Boote KJ 1998 Elevated CO_2_ and water deficit effects on photosynthesis, ribulose bisphosphate carboxylase-oxygenase, and carbohydrate metabolism in rice. Physiologia Plantarum 103, 327–339.

[CIT0049] Wanek W Heintel S Richter A 2001 Preparation of starch and other carbon fractions from higher plant leaves for stable carbon isotope analysis. Rapid Communications in Mass Spectrometry 15, 1136–1140. 11445894 10.1002/rcm.353

[CIT0050] Wegener F Beyschlag W Werner C 2010 The magnitude of diurnal variation in carbon isotopic composition of leaf dark respired CO_2_ correlates with the difference between δ^13^C of leaf and root material. Functional Plant Biology 37, 849–858.

[CIT0051] Werner C Gessler A 2011 Diel variations in the carbon isotope composition of respired CO_2_ and associated carbon sources: a review of dynamics and mechanisms. Biogeosciences 8, 2437–2459.

[CIT0052] Werner C Hasenbein N Maia R Beyschlag W Maguas C 2007 Evaluating high time-resolved changes in carbon isotope ratio of respired CO_2_ by a rapid in-tube incubation technique. Rapid Communications in Mass Spectrometry 21, 1352–1360. 17348086 10.1002/rcm.2970

[CIT0053] Werner C Wegener F Unger S Nogues S Priault P 2009 Short-term dynamics of isotopic composition of leaf-respired CO_2_ upon darkening: measurements and implications. Rapid Communications in Mass Spectrometry 23, 2428–2438. 19603472 10.1002/rcm.4036

[CIT0054] Werner RA Brand WA 2001 Referencing strategies and techniques in stable isotope ratio analysis. Rapid Communications in Mass Spectrometry 15, 501–519. 11268135 10.1002/rcm.258

[CIT0055] Werner RA Bruch BA Brand WA 1999 ConFlo III—an interface for high precision δ^13^C and δ^15^N analysis with an extended dynamic range. Rapid Communications in Mass Spectrometry 13, 1237–1241. 10407304 10.1002/(SICI)1097-0231(19990715)13:13<1237::AID-RCM633>3.0.CO;2-C

[CIT0056] Werner RA Buchmann N Siegwolf RTW Kornexl BE Gessler A 2011 Metabolic fluxes, carbon isotope fractionation and respiration—lessons to be learned from plant biochemistry. New Phytologist 191, 10–15. 21521226 10.1111/j.1469-8137.2011.03741.x

[CIT0057] Zeeman MJ Werner RA Eugster W Siegwolf RTW Wehrle G Mohn J Buchmann N 2008 Optimization of automated gas sample collection and isotope ratio mass spectrometric analysis of δ^13^C of CO_2_ in air. Rapid Communications in Mass Spectrometry 22, 3883–3892. 18988208 10.1002/rcm.3772

