# Supplementary material for: Malate as a key carbon source of leaf dark-respired CO2 across different environmental conditions in potato plants
Source: J Exp Bot. 2015 Jul 2;66(19):5769–81. doi: 10.1093/jxb/erv279 (PMC4566975; doi:10.1093/jxb/erv279)
Supplement: Supplementary Data [file supp_66_19_5769__index.html]

Malate as a key carbon source of leaf dark-respired CO2 across different environmental conditions in potato plants — Malate as a key carbon source of leaf dark-respired CO2 across different environmental conditions in potato plants — Supplementary Data 

# Malate as a key carbon source of leaf dark-respired CO2 across different environmental conditions in potato plants

## Supplementary Data

Data files

- Supplementary Data - Supplementary Data
