# Supplementary material for: Malate as a key carbon source of leaf dark-respired CO2 across different environmental conditions in potato plants
Source: J Exp Bot. 2015 Jul 2;66(19):5769–81. doi: 10.1093/jxb/erv279 (PMC4566975; doi:10.1093/jxb/erv279)
Supplement: Supplementary Data [file supp_erv279_jexbot145938_file001.pdf]

Daily regressions between leaf dark−respired CO2 and potential respiratory carbon sources

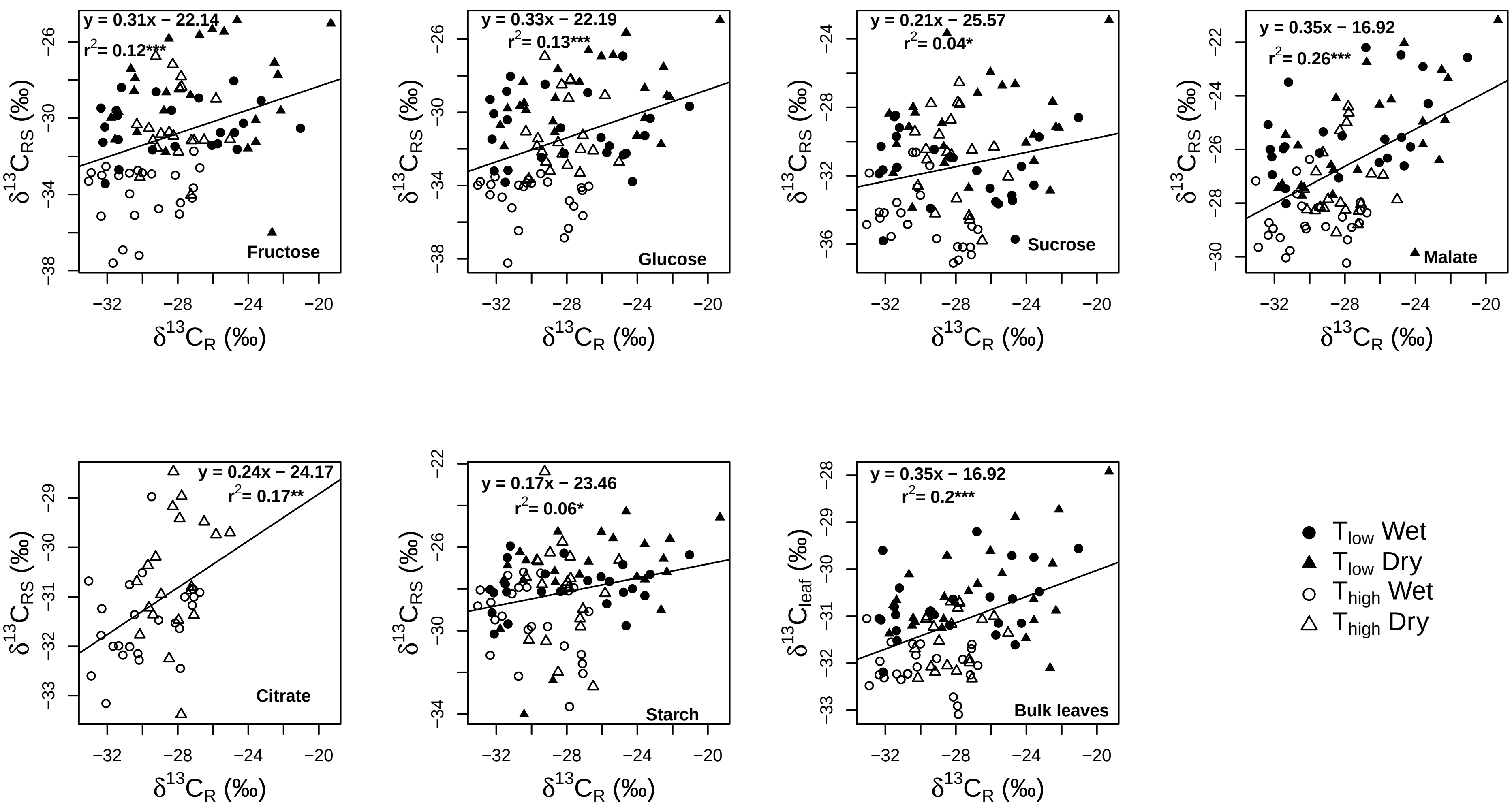

Daytime regressions between leaf dark−respired CO2 and potential respiratory carbon sources

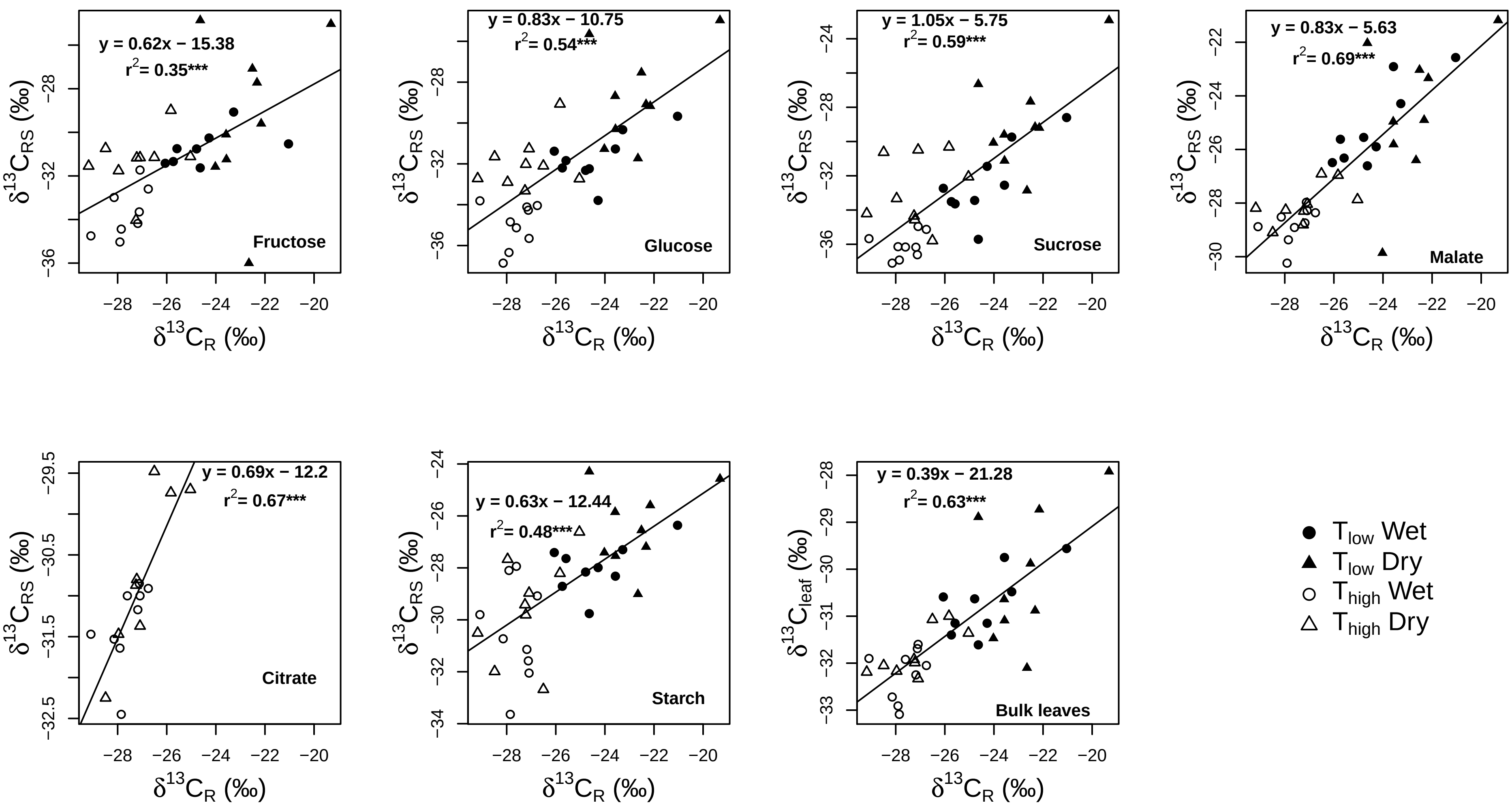

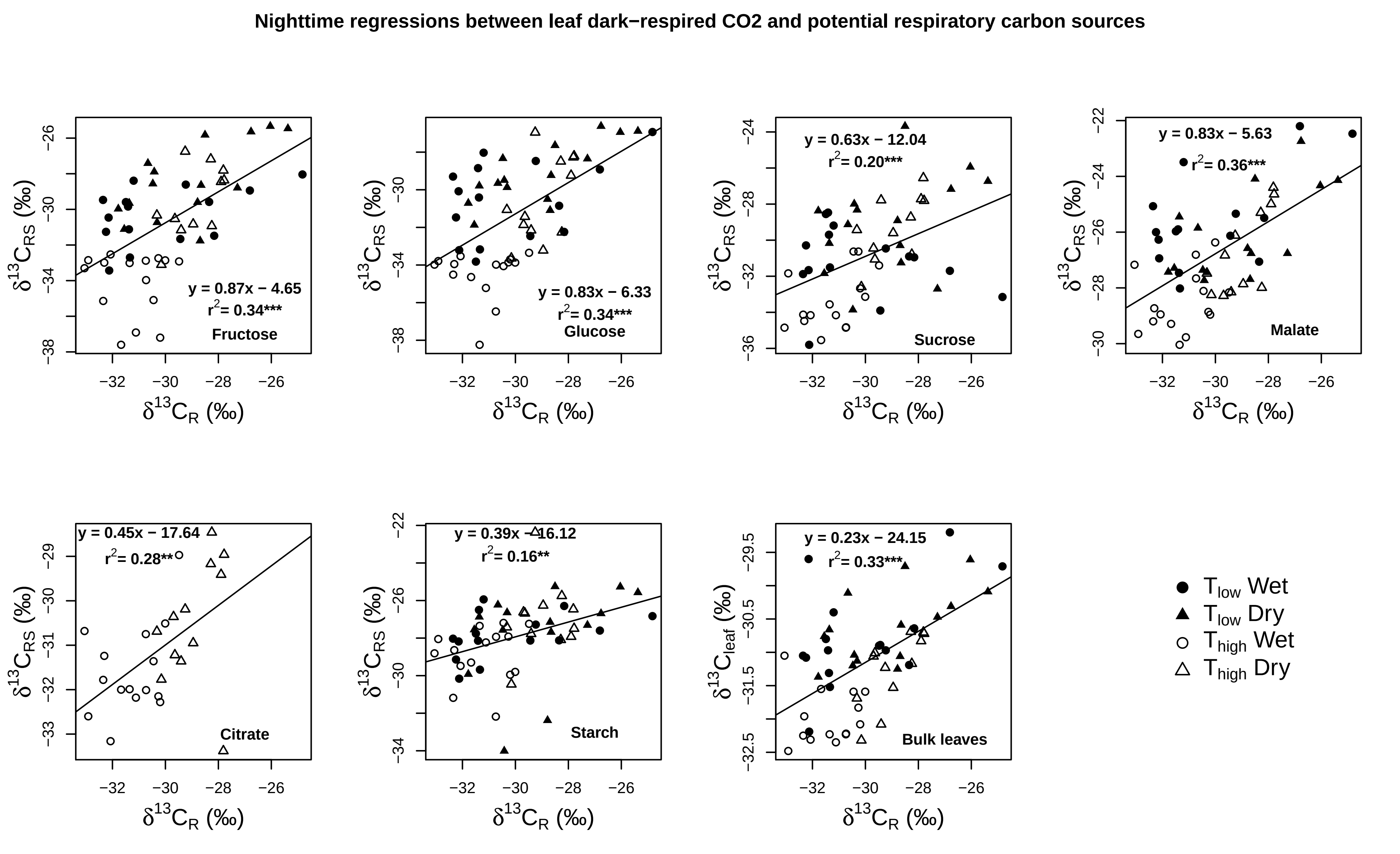

Nighttime regressions between leaf dark−respired CO2 and potential respiratory carbon sources
y = 0.87x − 4.65
r2= 0.34***
Fructose
y = 0.83x − 6.33
r2= 0.34***
Glucose
y = 0.63x − 12.04
r2= 0.20***
Sucrose
y = 0.83x − 5.63
r2= 0.36***
Malate
y = 0.45x − 17.64
r2= 0.28**
Citrate
y = 0.39x − 16.12
r2= 0.16**
Starch
y = 0.23x − 24.15
r2= 0.33***
Bulk leaves
δ13CRS (‰)
δ13Cleaf (‰)
δ13CR (‰)
Tlow Wet
Tlow Dry
Thigh Wet
Thigh Dry
